# Comparative Efficacy of Video Games Versus Midazolam in Reducing Perioperative Anxiety in Pediatric Patients: Systematic Review and Meta-Analysis

**DOI:** 10.2196/67007

**Published:** 2025-03-10

**Authors:** Ziyue Luo, Sisi Deng, Ruihao Zhou, Ling Ye, Tao Zhu, Guo Chen

**Affiliations:** 1 Department of Anesthesiology, National Clinical Research Center for Geriatrics, West China Hospital, Sichuan University, No. 37 Guoxue Lane, Wuhou District, Sichuan Province, Chengdu, 610041, China, 86 028-85423593; 2 Department of Pain Management, National Clinical Research Center for Geriatrics, West China Hospital, Sichuan University, Chengdu, China

**Keywords:** video games, midazolam, perioperative period, anxiety, meta-analysis, pediatric patients

## Abstract

**Background:**

Pediatric patients undergoing surgery frequently experience significant anxiety, which can result in adverse effects such as prolonged sedation and behavioral changes associated with pharmacological interventions such as oral midazolam. Video games offer a nonpharmacological distraction method that shows promise in alleviating procedural anxiety without significant adverse effects. However, the effectiveness of video games compared to midazolam in managing perioperative anxiety remains uncertain.

**Objective:**

This study aimed to evaluate the effectiveness of video game interventions in reducing perioperative anxiety in pediatric patients undergoing general anesthesia.

**Methods:**

We conducted a comprehensive search across PubMed, Embase, Web of Science, and the Cochrane Library, supplemented by reference screening. Primary outcomes included anxiety levels assessed during parent separation and mask induction procedures, while secondary outcomes encompassed emergence delirium, postoperative behavior, and length of stay in the postanesthesia care unit (PACU). The risk of bias was assessed using the Risk of Bias 2 scale. Data were synthesized descriptively and through meta-analysis, with the certainty of the evidence evaluated using the Grading of Recommendations Assessment, Development, and Evaluation (GRADE) criteria.

**Results:**

Six randomized controlled trials involving 612 participants were included in the analysis. Children who participated in video game interventions reported significantly lower anxiety levels during parent separation (standardized mean difference, SMD −0.31, 95% CI −0.50 to −0.12; *P*=.001), with high certainty, and during mask induction (SMD −0.29, 95% CI −0.52 to −0.05; *P*=.02), with moderate certainty, compared to those receiving oral midazolam. Additionally, significant differences in postoperative behavior changes in children were observed compared to oral midazolam (SMD −0.35, 95% CI −0.62 to −0.09; *P*=.008). Children in the video game intervention groups also had a shorter length of stay in the PACU (mean difference, MD −19.43 min, 95% CI −31.71 to −7.16; *P*=.002). However, no significant differences were found in emergence delirium (MD −2.01, 95% CI −4.62 to 0.59; *P*=.13).

**Conclusions:**

Video game interventions were more effective than midazolam in reducing perioperative anxiety among pediatric patients, improving postoperative behavior, and shortening the length of stay in the PACU. However, video games alone did not outperform midazolam in managing emergence delirium. Further high-quality research is needed for more conclusive results.

## Introduction

### Background

Perioperative anxiety is a significant concern for pediatric patients undergoing surgery [1]. This anxiety, characterized by worry, nervousness, or unease about uncertain outcomes, can lead to various negative effects, including increased preoperative distress, postoperative pain, and longer recovery times [2]. Research shows that high levels of preoperative anxiety are linked to greater postoperative pain and higher analgesic consumption [3], delayed hospital discharge [4], and the emergence of negative behavioral changes [5], such as nightmares, separation anxiety, and increased fear of medical procedures. Therefore, effectively managing perioperative anxiety is crucial for improving surgical outcomes and enhancing overall patient well-being.

Traditional methods for managing perioperative anxiety often rely on pharmacological interventions, with oral midazolam being a commonly used anxiolytic agent. Administered at a typical dosage of 0.25 to 0.5 mg/kg, midazolam is effective in reducing anxiety in children, inducing sedation and anxiolysis within 20 to 30 minutes [6,7]. Studies have shown that midazolam premedication can significantly improve cooperation during anesthesia induction and decrease postoperative behavioral disturbances [8,9]. However, the use of pharmacological agents is not without drawbacks. Potential adverse effects of midazolam include respiratory depression [10], paradoxical reactions [11], prolonged recovery periods [12], and postoperative cognitive dysfunction [13]. These concerns highlight the need to explore alternative, nonpharmacological interventions that can effectively manage perioperative anxiety without adverse effects.

In recent years, there has been growing interest in nonpharmacological interventions for managing anxiety in children undergoing surgery, such as music [14], clown doctors [15], preoperative preparation videos [16], virtual reality tools [17], augmented reality tools [18], and video games [19]. Video games, in particular, have been shown to be engaging and effective in reducing anxiety levels in various medical contexts [20]. A study indicated that video games can decrease both pain and anxiety in pediatric surgery patients [18]. Their interactive and immersive nature captivates children’s attention, providing a sense of control and normalcy in a potentially intimidating hospital environment. Patel et al [21] reported that distraction with hand-held video games significantly reduced preoperative anxiety levels compared to standard care. Additionally, video games offer the benefits of no adverse effects and enhancing patient cooperation during medical procedures.

The potential benefits of video games in clinical settings extend beyond distraction. They can also enhance patient engagement and compliance [22], reduce the need for sedative medications [15], and improve overall patient satisfaction with the surgical experience [23]. The versatility and appeal of video games make them a promising tool for anxiety management, warranting a systematic comparison with traditional pharmacological treatments such as oral midazolam. Given their increasing popularity and potential benefits, it is essential to compare their efficacy against traditional pharmacological treatments such as oral midazolam.

### Objectives

The primary objective of this systematic review and meta-analysis is to compare the efficacy of video games and midazolam in reducing perioperative anxiety in pediatric patients during critical moments, such as parent separation and mask induction. Additionally, we conducted a subgroup analysis of midazolam dosages related to anxiety. Furthermore, the review aims to assess the impact of these interventions on secondary outcomes, including emergence delirium, postoperative behavior, and length of stay in the postanesthesia care unit (PACU). This is the first systematic review and meta-analysis to evaluate the effects of video games on outcomes related to managing perioperative anxiety in children.

## Methods

This systematic review follows the PRISMA (Preferred Reporting Items for Systematic Reviews and Meta-Analyses) 2020 guidelines (Checklist 1) [24]. Additionally, we formally registered this systematic review and meta-analysis with the International Prospective Register of Systematic Reviews (PROSPERO, CRD42023486085).

### Search Strategy

We conducted a comprehensive literature search in PubMed, Embase, Web of Science, and the Cochrane Library up to June 30, 2024. The search strategy included Medical Subject Headings and text words related to “video game,” “midazolam,” and “anxiety.” Search terms were tailored for each database to ensure comprehensiveness. The detailed search strategies for each database are provided in Multimedia Appendix 1. Furthermore, the reference lists of the included studies were scrutinized for articles not initially identified in the primary search. There were no restrictions on the publication date, but articles were limited to those published in English.

### Study Eligibility Criteria

Inclusion criteria for this study were as follows: (1) Population: Children (≤18 years old) undergoing surgical procedures under general anesthesia. (2) Intervention: Video games utilized as a perioperative intervention to alleviate anxiety. (3) Comparators: Midazolam as a perioperative intervention for anxiety relief. (4) Outcomes: Primary outcomes included anxiety levels during parent separation and mask induction, while secondary outcomes comprised emergence delirium, postoperative behavior, and length of stay in the PACU. Various measurement methods were accepted, including self-report, proxy report, and observation. (5) Study design: Only randomized controlled trials were considered eligible for inclusion.

The exclusion criteria were as follows: (1) studies not involving pediatric surgical patients under general anesthesia; (2) patients who received anxiolytic premedication or had cognitive impairments such as psychiatric disorders or autism; and (3) nonrandomized controlled trials, reviews, meta-analyses, and single case reports.

### Study Selection and Data Extraction

We combined the search results from the four databases and removed duplicate articles. Two investigators (ZYL and SSD) independently screened titles/abstracts and full texts using a shared spreadsheet (Microsoft Excel). To implement the double-blind process, each reviewer maintained a separate decision sheet. Discrepancies in evaluations triggered a re-examination of the conflicting articles. The investigators then discussed the articles’ eligibility for inclusion or exclusion. If disagreements persisted, a third senior author was consulted for resolution during the systematic review.

For the review, two investigators (ZYL and SSD) extracted the data from the eligible articles. The data included the name of the first author, publication year, participant information (sample size and age), study location (country), intervention and control group details, and instruments and time points of outcome assessment. Disagreements between reviewers were resolved through discussion. In cases of missing or unclear data, the study authors were contacted twice via email.

### Study Quality Assessment

The revised Cochrane risk of bias tool for randomized trials was employed to analyze the risk of bias in randomized studies included in this review, as recommended elsewhere [25]. The tool comprises five domains with different questions: (1) bias arising from the randomization process; (2) bias due to deviations from intended interventions; (3) bias due to missing outcome data; (4) bias in measurement of the outcome; and (5) bias in selection of the reported result. The risk of bias in each domain was categorized into three levels: “low risk of bias,” “some concerns,” and “high risk of bias.”

### Data Synthesis and Analysis

For the meta-analysis, eligible studies were analyzed to combine results, ensuring clinical and methodological homogeneity of the intervention and follow-up period. We utilized Review Manager 5.4 (The Cochrane Collaboration) for conducting the meta-analysis and heterogeneity testing. Where necessary, data transformations were performed prior to analysis, including converting standard errors of the mean (SEMs) to standard deviations (SDs) using the formula SD=SEM×$\sqrt{n}$ and transforming medians (IQRs) to means (SDs) with the method given by Wan et al [26], assuming normal or log-normal distributions, to ensure compatibility for meta-analysis. For outcome-specific effect measures, standardized mean differences (SMDs) were calculated for perioperative anxiety and postoperative behavioral outcomes due to heterogeneity in measurement scales across studies, while mean differences (MDs) were used for emergence delirium and length of stay in the PACU, as these outcomes shared consistent measurement units; all effect estimates are reported with 95% CIs. To enhance clinical interpretability, SMDs were converted back to the original measurement scale by multiplying them with the pooled standard deviation (SD_pooled_) of the included studies. Forest plots were generated to visualize the results. Given the anticipated clinical and methodological diversity across studies, including variations in participant characteristics, surgical procedures, cultural contexts, and intervention protocols, we selected a random-effects model a priori for all meta-analyses. Heterogeneity among the studies was assessed using the *χ*^2^ test, with a significance threshold set at *P*<.10 [27]. Additionally, the *I^²^* test was employed to quantify the extent of variability, categorizing it as 0%‐40% as potentially unimportant, 30%‐60% as moderate, 50%‐90% as substantial, and 75%‐100% as considerable, taking into account the magnitude and direction of effects as well as the strength of the evidence [28] . Statistical significance was determined through two-tailed tests, with a threshold of *P*<.05. Additionally, we conducted a subgroup analysis of midazolam dosages related to perioperative anxiety.

We utilized the Grading of Recommendations Assessment, Development, and Evaluation (GRADE) framework to assess the evidence level of the included outcomes [29]. This evaluation was performed with GRADEpro software (McMaster University and Evidence Prime Inc). The following five domains of the GRADE criteria were analyzed: methodological limitations (risk of bias), inconsistency, indirectness, imprecision, and publication bias. Each domain involves a qualitative assessment of the evidence for each outcome, allowing the classification of confidence in the estimated effects as high, moderate, low, or very low.

## Results

### Search Results and Selection

**Figure 1.**
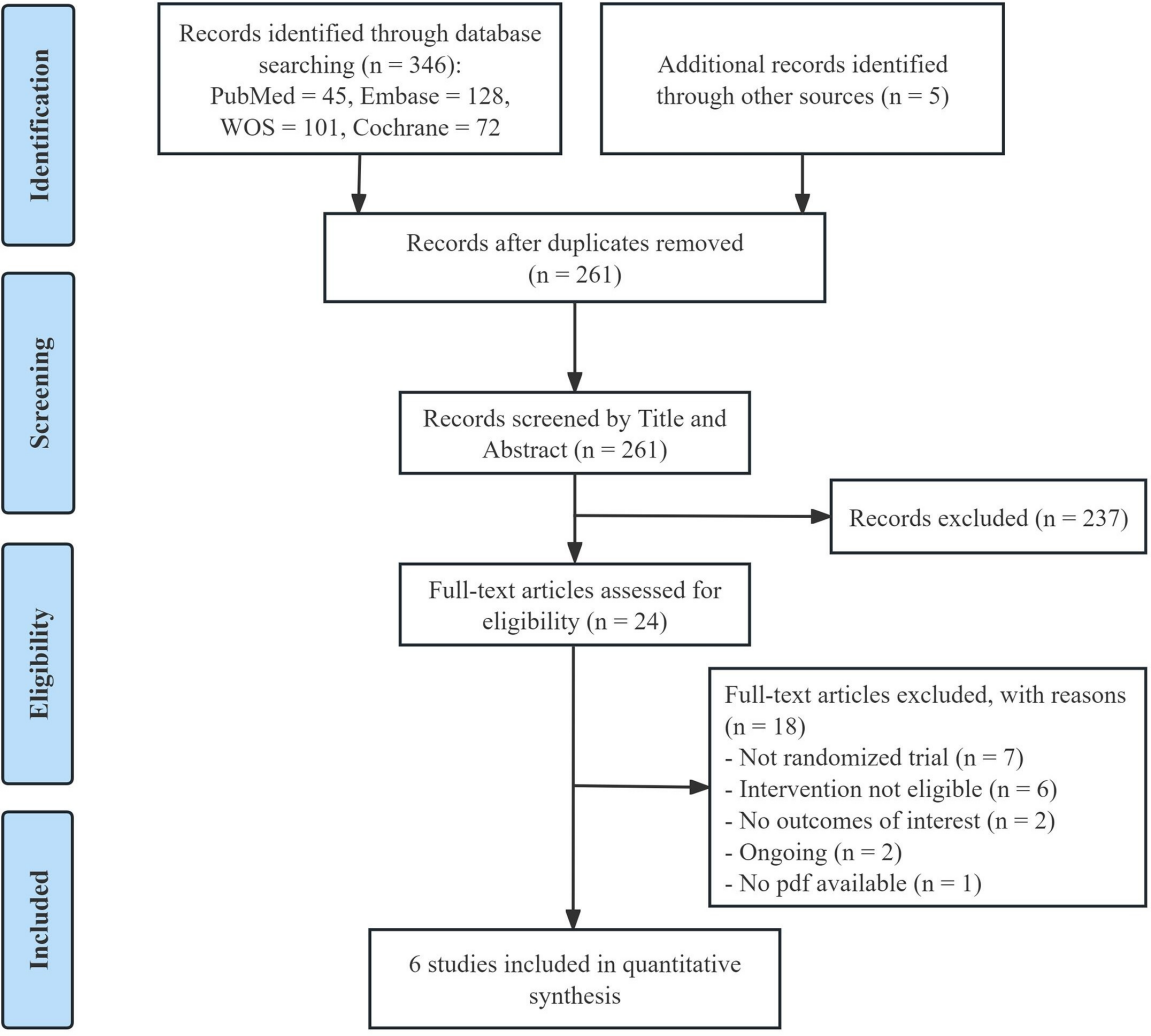
Study flow diagram of the search strategy and included studies.

We conducted a comprehensive search across four databases, identifying a total of 346 articles. After removing duplicates, 256 studies remained for screening. Following a review of titles and abstracts, irrelevant studies were excluded, leaving 24 full-text articles for further assessment. Ultimately, 6 studies met the inclusion criteria and were included in our meta-analysis [21,23,30-33]. The detailed screening process is presented in Figure 1.

### Characteristics and Results of Individual Studies

The meta-analysis encompasses 6 studies from France (2 studies) and the United States (4 studies) that assessed interventions aimed at reducing preoperative anxiety and managing postoperative behavior among pediatric patients. The patient cohort, totaling 612 subjects aged 3 to 12 years, was predominantly classified as having an American Society of Anesthesiologists score of I-II. These studies compared the effectiveness of age-appropriate video games (316 participants) against standard midazolam dosages of 0.3 mg/kg or 0.5 mg/kg (296 participants) administered before anesthesia. Anxiety levels were systematically assessed at multiple time points utilizing either the Modified Yale Preoperative Anxiety Scale (mYPAS) or its abbreviated version, the mYPAS-Short Form. The mYPAS includes 27 items related to activity, arousal, vocalization, dependence on parents, and emotional status. The total score ranges from 23 to 100, with scores above 30 indicating anxiety and scores above 40 indicating high anxiety [34]. Postoperative behavioral outcomes were evaluated through the implementation of either the posthospital behavior questionnaire (PHBQ) or the posthospitalization behavior change questionnaire for ambulatory surgery. The PHBQ comprises 27 items among 6 subscales (general anxiety and regression, separation anxiety, eating disturbance, aggression toward authority, apathy/withdrawal, and anxiety about sleep) [35]. Caregivers were also given the response option of not applicable. Furthermore, the incidence of emergence delirium was quantitatively measured using the validated pediatric anesthesia emergence delirium (PAED) scale. The PAED Scale [36] consists of 5 items scored from 0 to 4 (with 3 reverse-scored items). The 5 items relate to the observation of eye contact with caregivers, purposeful actions, awareness of surroundings, restlessness, and inconsolability. Detailed characteristics and results of these studies are summarized in Multimedia Appendix 2.

### Risk of Bias Assessment

**Figure 2.**
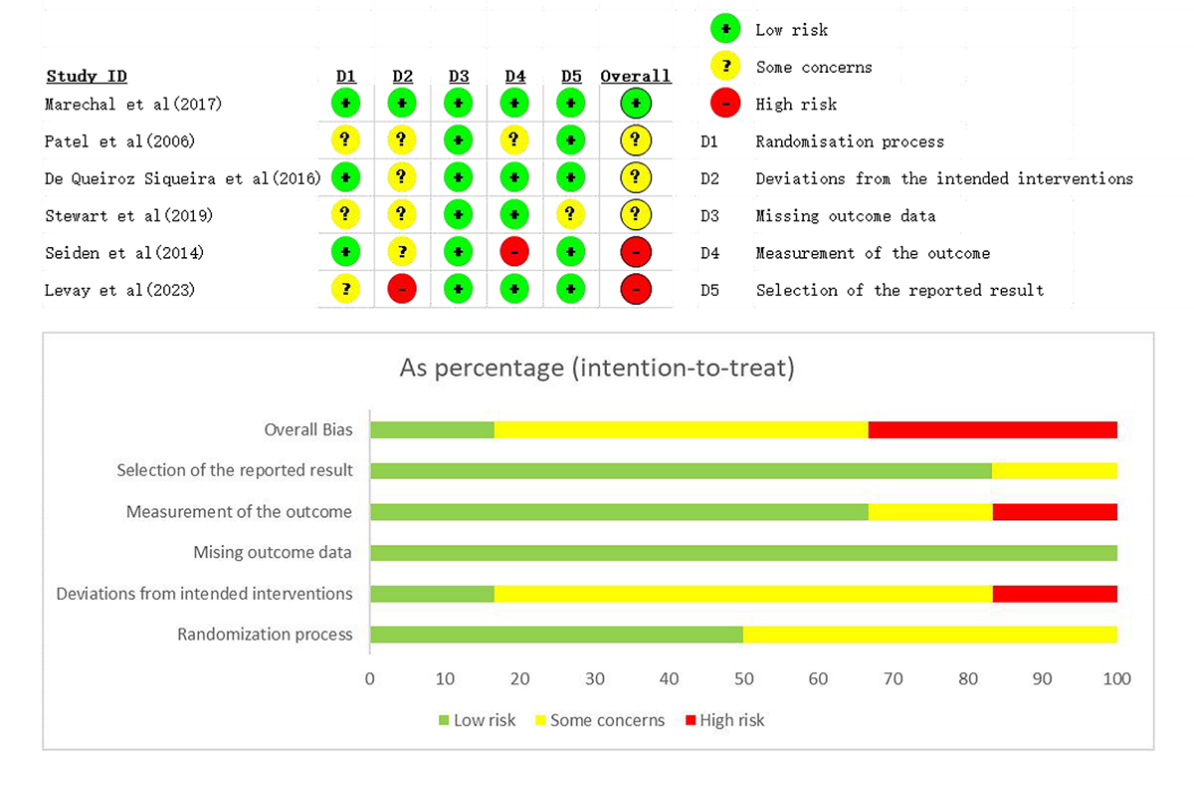
Risk of bias assessment of the included studies [21,23,30-33] using version 2 of the Cochrane risk-of-bias tool for randomized trials tool.

We assessed the risk of bias for the 6 included randomized controlled trials using the RoB 2 tool (version 2 of the Cochrane risk-of-bias tool for randomized trials). The analysis revealed that 1 article was rated as low risk [31], 3 articles exhibited some concerns and were thus categorized as medium risk [21,30,32], and 2 articles were classified as high risk [23,33]. The concerns predominantly stemmed from issues such as inadequate blinding of participants and deviations from intended interventions. Specifically, the 2 studies identified as having a high risk of bias faced significant issues related to deviations from intended interventions and inaccuracies in the measurement of outcomes [23,33]. These findings are detailed in Figure 2.

### Meta-Analysis

#### Pediatric Anxiety

**Figure 3.**
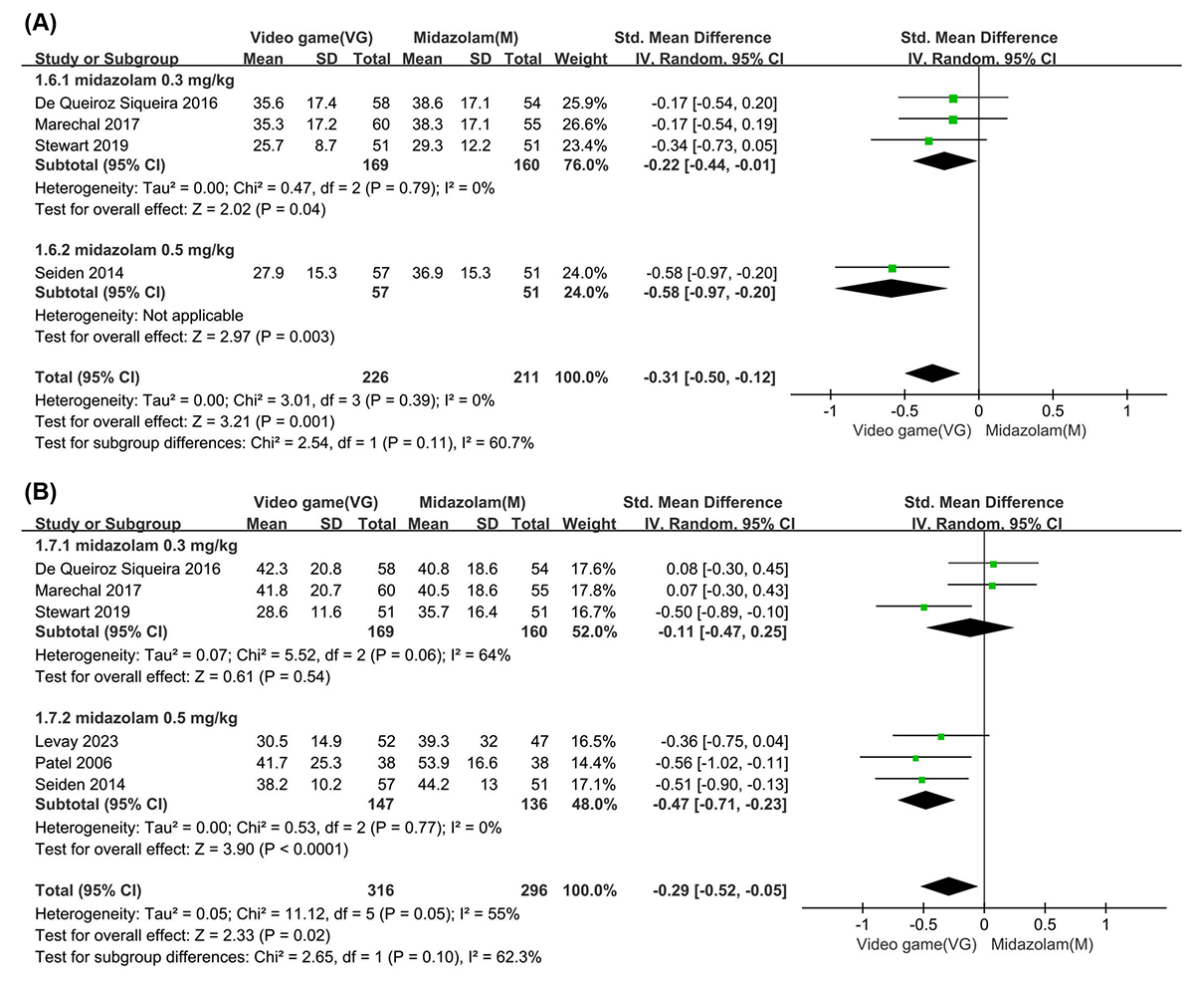
Forest plot comparing anxiety levels: video game intervention versus midazolam during (**A**) parent separation [23,30-32] and (**B**) mask induction [21,23,30-33].

Data on pediatric anxiety at the time of parent separation were obtained from 4 studies, totaling 226 participants [23,30-32]. Overall, children who engaged in video games exhibited significantly lower anxiety levels compared to those in the control group, showing a statistically significant effect (SMD −0.31, 95% CI −0.50 to −0.12; *P*=.001) with high certainty (Table 1). According to the Cohen criteria [37], this corresponds to a small-to-moderate effect size. After conversion to the original anxiety scale, the estimated MD was −5.23 points. Additionally, no significant heterogeneity was found among the included studies (*I^²^*=0%; *P*=.39). Subgroup analysis further confirmed that children playing video games reported lower anxiety levels in both the 0.3 mg/kg midazolam subgroup (SMD −0.22, 95% CI −0.44 to −0.01; *P*=.04), corresponding to a small effect size and translating to a MD of −3.81 points on the original anxiety scale, and the 0.5 mg/kg midazolam subgroup (SMD −0.58, 95% CI −0.97 to −0.20; *P*=.003), corresponding to a moderate-to-large effect size and equating to a MD of −8.98 points. The heterogeneity test for subgroup differences indicated some variability (*I²*=60.7%; *P*=.11) (Figure 3A).

Data on pediatric anxiety during mask induction were collected from 6 studies involving 316 participants [21,23,30-33]. The results indicated a significant difference in anxiety reduction between children playing video games and those receiving midazolam (SMD −0.29, 95% CI −0.52 to −0.05; *P*=.02), indicating a small-to-moderate effect, with an estimated MD of −5.73 points after conversion to the original anxiety scale and moderate certainty (Table 1). However, moderate heterogeneity was observed among the studies (*I^²^*=55%; *P*=.05). Subgroup analysis revealed no significant difference in the 0.3 mg/kg midazolam subgroup (SMD −0.11, 95% CI −0.47 to 0.25; *P*=.54), indicating a very small effect, with an estimated MD of −2.17 points. In contrast, a statistically significant effect was found in the 0.5 mg/kg midazolam subgroup (SMD −0.47, 95% CI −0.71 to −0.23; *P*<.001), indicating a moderate-to-large effect, equating to an estimated MD of −9.28 points. The heterogeneity test for subgroup differences indicated some variability (*I^²^*=62.3%; *P*=.10) (Figure 3B).

**Table 1.** Result of assessment of certainty of evidence for all outcomes of the use of video games compared to midazolam in children in the operation room.

Outcomes	Relative effect (95% CI)^a^	No. of Participants (no. of studies)	Quality of the evidence (GRADE^b^)
Anxiety T1^c^	SMD^d^ −0.31 (−0.5 to −0.12)	437 (4 studies)	⊕⊕⊕⊕ high^e^
Anxiety T2^f^	SMD −0.29 (−0.52 to −0.05)	612 (6 studies)	⊕⊕⊕⊝ moderate^g^^h^
Emergence delirium	MD^i^ −2.01 (−4.62 to 0.59)	309 (3 studies)	⊕⊝⊝⊝ very low^j^^k^^l^
Postoperative Behavior	SMD −0.35 (−0.62 to −0.09)	227 (3 studies)	⊕⊕⊕⊕ high
Length of stay in the PACU^m^	MD −19.43 minutes (−31.71 to −7.16)	309 (3 studies)	⊕⊕⊕⊕ high

a CI: confidence interval.

b Grading of Recommendations Assessment, Development, and Evaluation Working Group grades of evidence.

c Anxiety T1: anxiety at the time of parent separation.

d SMD: standardized mean difference.

e High quality: Further research is very unlikely to change our confidence in the estimate of effect.

f Anxiety T2: anxiety at the time of mask induction.

g Moderate quality: Further research is likely to have an important impact on our confidence in the estimate of effect and may change the estimate.

h Downgraded by 1 level due to moderate concerns about inconsistency, including notable heterogeneity in effect estimates across trials, reflecting variations in both the magnitude and direction of effect sizes (50%< I²< 75%).

i MD: mean difference.

j Very low quality: We are very uncertain about the estimate.

k Downgraded by 1 level due to significant concerns about imprecision, the confidence interval suggests the possibility of a null effect or benefit for either intervention.

l Downgraded by 2 levels due to serious concerns about inconsistency, with substantial heterogeneity in effect estimates across trials (I²≥ 75%).

m PACU: postanesthesia care unit.

### Emergence Delirium

**Figure 4.**
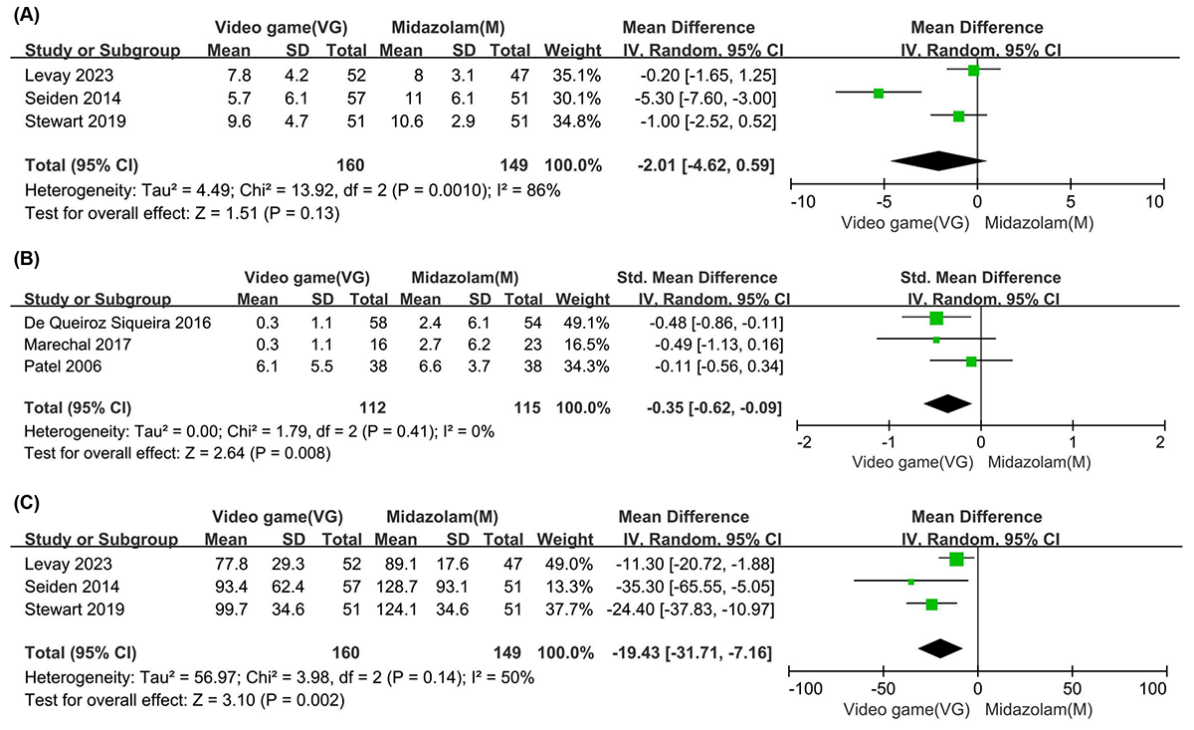
Forest plot comparing video game intervention and midazolam: (**A**) emergence delirium [23,32,33], (**B**) postoperative behavior [21,30,31], and (**C**) length of stay in the postanesthesia care unit (PACU) [23,32,33].

Data on emergence delirium were collected from 3 studies, comprising a total of 160 participants [23,32,33]. The results showed no significant differences between children who played video games and those who received midazolam (MD −2.01, 95% CI −4.62 to 0.59; *P*=.13), with a very low certainty (Table 1). Additionally, substantial heterogeneity was observed (*I²*=86%; *P*=.001) (Figure 4A).

### Postoperative Behavior

Data on postoperative behavior were available from 3 studies, comprising a total of 112 participants [21,30,31]. A significant difference was found between the video game group and the midazolam group (SMD −0.35, 95% CI −0.62 to −0.09; *P*=.008), corresponding to a small-to-moderate effect size. After conversion to the original postoperative behavior scale, this equates to an estimated MD of −1.59 points. The quality of evidence was classified as high (Table 1). No substantial heterogeneity was observed (*I^²^*=0%; *P*=.41) (Figure 4B).

### Length of Stay in the PACU

Data on the length of stay in the PACU were available from 3 studies, totaling 160 participants [23,32,33]. Children who received video game interventions had significantly shorter PACU stays (MD −19.43 min, 95%CI −31.71 to −7.16 min; *P*=.002). The quality of the evidence was rated as high (Table 1), and moderate heterogeneity was observed across the studies (*I^²^*=50%; *P*=.14) (Figure 4C).

## Discussion

### Principal Findings

This systematic review and meta-analysis assessed the effectiveness of video games compared to midazolam in reducing preoperative anxiety in pediatric patients. We identified 6 studies involving 612 children, which produced mixed outcomes. Our meta-analysis indicates that video games were more effective than midazolam in reducing anxiety during both parental separation and mask induction, critical moments of heightened stress for children undergoing surgery.

At the time of parental separation, the subgroup analysis demonstrated that video games outperformed midazolam at both the 0.3 mg/kg and 0.5 mg/kg doses, with a more pronounced effect observed in the 0.5 mg/kg group. This finding may be linked to the challenges of administering oral medications to pediatric patients, as well as midazolam’s potential paradoxical effects, which can lead to increased agitation instead of calming the child. Additionally, factors such as the age and mood of the children may contribute to this variability [38]. One study found that approximately 14% of children who received oral midazolam prior to surgery still exhibited extreme anxiety and lack of compliance during anesthesia induction [39].

At the time of mask induction, a significant difference was found between video games and midazolam, suggesting that video games may be more effective in reducing anxiety during this phase. However, the subgroup analysis revealed no significant differences between the two interventions at the 0.3 mg/kg dose. This lack of difference may be due to the insufficient potency of this lower dose, which might not effectively reduce anxiety in children [40]. Additionally, the engaging nature of video games could provide enough distraction to manage anxiety, resulting in comparable outcomes to midazolam [19]. In contrast, at the 0.5 mg/kg dose, video games demonstrated superiority over midazolam. This superiority may be attributed to the potential for paradoxical reactions to midazolam, which can lead to increased agitation in some children, as well as the higher potency of this dose introducing variability in effectiveness [41]. These factors, along with individual differences in responses to medication, likely contributed to the greater effectiveness of video games during this critical moment.

Significant differences were noted in postoperative behavior and length of stay in the PACU, suggesting that video games may offer an effective interactive distraction that helps children cope better in the postoperative period [19]. This engagement can serve to divert their attention from discomfort and anxiety associated with recovery, potentially leading to improved behavioral outcomes. The immersive nature of video games can facilitate a sense of control and agency, which is especially important for children facing medical procedures. Additionally, video games may promote relaxation and positive emotional states, further enhancing their ability to manage pain and anxiety [42]. Furthermore, the reduction in length of stay in the PACU observed in children receiving video game interventions suggests that these distractions may contribute to a more efficient recovery process. One possible mechanism is the attenuation of stress-related physiological responses, such as reduced sympathetic nervous system activation, which can facilitate faster stabilization of vital signs postoperatively. Moreover, by lowering perioperative anxiety and distress, video games may help mitigate the need for additional sedatives or analgesics, which could otherwise prolong PACU stays. Additionally, improved postoperative cooperation and reduced agitation may enable earlier discharge from the PACU, optimizing resource utilization in clinical settings. This highlights the potential for video game interventions not only to enhance patient experience but also to improve hospital workflow efficiency [43].

We found no significant differences between video games and midazolam regarding emergence delirium, indicating that nonpharmacological interventions may have limited effects on these outcomes. Several factors could contribute to this lack of difference, including the possibility that both interventions are similarly effective or that the nature of emergence delirium is such that it may not be easily alleviated by distractions alone [44]. Additionally, the context in which these interventions are applied may play a role, as factors such as individual patient characteristics [45] and the surgical environment [46] could influence outcomes. However, the evidence was classified as very low quality, and the substantial heterogeneity and inconsistency across studies weaken the strength of this conclusion, necessitating caution in interpretation. Further high-quality research is needed to confirm these findings and better understand the potential impact of nonpharmacological interventions on emergence delirium. Robust studies with larger sample sizes and standardized methodologies will be essential to draw more definitive conclusions.

### Limitations

The credibility of our findings is limited by several factors. High levels of heterogeneity in the type, duration, and frequency of video games used in the studies made direct comparisons challenging. Bias was also a concern, as many studies lacked blinding, which may have skewed the results. Additionally, the small sample sizes in several studies likely affected statistical power, further limiting the generalizability of our findings. The subjectivity of various assessment scales is noteworthy; for instance, Seiden [23] reported mean baseline mYPAS values ranging from 23 to 45, while Levay [33] reported scores from 23 to 65. This variability may arise from the fact that mYPAS or other assessments were often administered by staff members, introducing potential bias.

Furthermore, the limited number of studies highlights the need for more high-quality research to strengthen our conclusions. Additionally, our review did not consider the potential impact of caregiver anxiety. A systematic review indicates that parent’s and children’s experiences are closely interconnected, with caregiver anxiety potentially exacerbating a child’s anxiety and leading to long-term psychosocial effects, such as increased fear and guilt [47]. Future research should investigate the role of caregiver anxiety in pediatric surgical settings and explore effective interventions to alleviate it, ultimately enhancing the overall well-being of both children and their families during these critical experiences.

### Conclusions

In summary, video games offer an accessible, low-cost, and well-tolerated intervention for reducing perioperative anxiety in pediatric patients undergoing general anesthesia, making them a promising nonpharmacological alternative to midazolam in certain contexts. However, to achieve a more comprehensive approach to pediatric perioperative care, a multimodal strategy should be implemented. This approach would integrate both pharmacological and nonpharmacological interventions tailored to each child’s unique needs and specific clinical situation. Additionally, further high-quality, large-scale studies are essential to confirm these findings, reduce heterogeneity, and investigate the integration of caregiver anxiety management into pediatric perioperative protocols. By addressing the emotional needs of both children and their caregivers, we can enhance overall outcomes and better support families during surgical experiences.

## Supplementary material

10.2196/67007 Multimedia Appendix 1 Search strategy.

10.2196/67007 Multimedia Appendix 2 Characteristics of the studies included in the review.

10.2196/67007 Checklist 1 The PRISMA (Preferred Reporting Items for Systematic Reviews and Meta-Analyses) checklist.

## Abbreviations

GRADE: Grading of Recommendations, Assessment, Development, and Evaluation
MD: mean difference
mYPAS: modified Yale Preoperative Anxiety Scale
PACU: postanesthesia care unit
PAED: pediatric anesthesia emergence delirium
PHBQ: posthospital behavior questionnaire
PRISMA: Preferred Reporting Items for Systematic Reviews and Meta-Analyses
RoB 2: version 2 of the Cochrane risk-of-bias tool for randomized trials
SMD: standardized mean difference
