# Supplementary material for: Comparative Efficacy of Video Games Versus Midazolam in Reducing Perioperative Anxiety in Pediatric Patients: Systematic Review and Meta-Analysis
Source: JMIR Serious Games. 2025 Mar 10;13:e67007. doi: 10.2196/67007 (PMC11913429; doi:10.2196/67007)
Supplement: Multimedia Appendix 1 [file games-v13-e67007-s001.docx]

**Multimedia Appendix 1.** Formal search strategy.

| **PubMed** | | |
| --- | --- | --- |
| #1 | Video Games[MeSH Terms] OR Games, Video[Title/Abstract] OR Game, Video[Title/Abstract] OR Video Game[Title/Abstract] OR Computer Games[Title/Abstract] OR Computer Game[Title/Abstract] OR Game, Computer[Title/Abstract] OR Games, Computer[Title/Abstract] OR distract[Title/Abstract] OR distractability[Title/Abstract] OR distractable[Title/Abstract] OR distracted[Title/Abstract] OR distracter[Title/Abstract] OR distracters[Title/Abstract] OR distractibility[Title/Abstract] OR distractible[Title/Abstract] OR distracting[Title/Abstract] OR distraction[Title/Abstract] OR distractional[Title/Abstract] OR distractions[Title/Abstract] OR distractive[Title/Abstract] OR distracts[Title/Abstract] | |
| #2 | midazolam[MeSH Terms] OR midazolam[Title/Abstract] OR midazolam s[Title/Abstract] OR midazolame[Title/Abstract] OR Dormicum[Title/Abstract] OR Ro 21-3981[Title/Abstract] OR Ro 213981[Title/Abstract] OR Ro 21 3981[Title/Abstract] | |
| #3 | General Surgery[MeSH Terms] OR Surgery, General[Title/Abstract] OR Surgery[Title/Abstract] OR Surgical Procedures, Operative[Title/Abstract] OR Operative Procedures[Title/Abstract] OR Operative Procedure[Title/Abstract] OR Procedure, Operative[Title/Abstract] OR Procedures, Operative[Title/Abstract] OR Surgical Procedure, Operative[Title/Abstract] OR Operative Surgical Procedures[Title/Abstract] OR Procedure, Operative Surgical[Title/Abstract] OR Procedures, Operative Surgical[Title/Abstract] OR Surgical Procedures[Title/Abstract] OR Procedure, Surgical[Title/Abstract] OR Procedures, Surgical[Title/Abstract] OR Surgical Procedure[Title/Abstract] OR Operative Surgical Procedure[Title/Abstract] OR Surgery, Ghost[Title/Abstract] OR Ghost Surgery[Title/Abstract] OR perioperative period[Title/Abstract] OR Period, Perioperative[Title/Abstract] OR Periods, Perioperative[Title/Abstract] OR Perioperative Periods[Title/Abstract] | |
| #4 | Child[MeSH Terms] OR Children[Title/Abstract] OR Boy[Title/Abstract] OR childhood[Title/Abstract] OR girl[Title/Abstract] OR infant[Title/Abstract] OR kid[Title/Abstract] OR pediatrics[Title/Abstract] OR preschool[Title/Abstract] OR school[Title/Abstract] OR toddler[Title/Abstract] OR Adolescents[Title/Abstract] OR Adolescence[Title/Abstract] OR adolescent[Title/Abstract] OR high school[Title/Abstract] OR juvenile[Title/Abstract] OR minor[Title/Abstract] OR prepubescent[Title/Abstract] OR prepuberty[Title/Abstract] OR pubescent[Title/Abstract] OR puberty[Title/Abstract] OR teen[Title/Abstract] OR teenager[Title/Abstract] OR underaged[Title/Abstract] OR youth[Title/Abstract] | |
| #5 | #1 AND #2 AND #3 AND #4 | |
| **Embase** | | |
| #1 | game*:ti,ab,kw OR distract*:ti,ab,kw | |
| #2 | 'midazolam'/exp OR 'ro 21-3981':ti,ab,kw OR dormicum:ti,ab,kw | |
| #3 | surg*:ti,ab,kw OR operat*:ti,ab,kw OR preop*:ti,ab,kw OR periop*:ti,ab,kw OR postop*:ti,ab,kw OR procedure*:ti,ab,kw | |
| #4 | Child*:ti,ab,kw OR boy:ti,ab,kw OR girl:ti,ab,kw OR infant:ti,ab,kw OR kid*:ti,ab,kw OR pediatrics:ti,ab,kw OR preschool:ti,ab,kw OR school:ti,ab,kw OR toddler:ti,ab,kw OR adolescen*:ti,ab,kw OR juvenile:ti,ab,kw OR minor:ti,ab,kw OR prepubescent:ti,ab,kw OR prepuberty:ti,ab,kw OR teen*:ti,ab,kw OR underaged:ti,ab,kw OR youth:ti,ab,kw | |
| #5 | #1 AND #2 AND #3 AND #4 | |
| **Web of Science** | | |
| #1 | | TS=( game* OR distract*) |
| #2 | | TS=( surg* OR operat* OR preop* OR period* OR procedure*) |
| #3 | | TS=( midazolam OR Dormicum OR Ro 21-3981) |
|  | | TS=( Child* OR boy OR girl OR infant OR kid* OR pediatrics:ti,ab,kw OR preschool OR school OR toddler OR adolescen* OR juvenile OR minor OR prepubescent OR prepuberty OR teen* OR underaged OR youth) |
| #4 | | #1 AND #2 AND #3 AND #4 |
| **Cochrane Library** | | |
| #1 | | MeSH descriptor: [Midazolam] explode all trees |
| #2 | | (ro 21 3981):ti,ab,kw OR (dormicum):ti,ab,kw OR (Midazolam):ti,ab,kw |
| #3 | | #1 OR #2 |
| #4 | | (surg* OR operat* OR preop* OR periop* OR postop* OR procedure*):ti,ab,kw |
| #5 | | (game* OR distract*):ti,ab,kw |
| #6 | | (Child* OR boy OR girl OR infant OR kid* OR pediatrics:ti,ab,kw OR preschool OR school OR toddler OR adolescen* OR juvenile OR minor OR prepubescent OR prepuberty OR teen* OR underaged OR youth):ti,ab,kw |
| #7 | | #3 and #4 and #5 and #6 |
