# Supplementary material for: Comparative Efficacy of Video Games Versus Midazolam in Reducing Perioperative Anxiety in Pediatric Patients: Systematic Review and Meta-Analysis
Source: JMIR Serious Games. 2025 Mar 10;13:e67007. doi: 10.2196/67007 (PMC11913429; doi:10.2196/67007)
Supplement: Multimedia Appendix 2 [file games-v13-e67007-s002.docx]

**Multimedia Appendix 2.** Characteristics of the studies included in the review.

| **Included studies** | **Country** | **Population** | **Participants** | **Intervention** | **Comparator** | **Outcomes** |
| --- | --- | --- | --- | --- | --- | --- |
| Marechal et al, 2017 | France | 4-10 years, ASA I-III | 115  (60 intervention and 55 control) | age-appropriate tablet games, 20 min before anaesthesia to be continued until loss of consciousness | midazolam 0.3 mg/kg p.o. or p.r., 20-30 minutes before anaesthesia | **Anxiety:** mYPAS: upon arrival to the ambulatory surgery ward, at separation from parents, at mask induction and when back in the ambulatory surgery ward  **Behavior:** PHBQ: at 1, 7 and 14 postoperative days |
| Patel et al, 2006 | America | 4–12 years, ASA I-II | 76  (38 intervention and 38 control) | a hand-held video game, at least 20 min before entering the operating room | midazolam 0.5 mg/kg p.o., at least 20 minutes before entering the operating room | **Anxiety:** mYPAS: prior to randomization or any intervention and at induction of anesthesia  **Behavior:** PHBQ: between 7 and 10 after surgery |
| De Queiroz Siqueira et al, 2016 | France | 4-10 years, NA | 112  (58 intervention and 54 control) | age-appropriate games, 20 min before anaesthesia to be continued until loss of consciousness | midazolam 0.3 mg/kg p.o. or p.r, 20-30 minutes before anaesthesia | **Anxiety:** mYPAS: at arrival at hospital, at separation from the parents and during induction  **Behavior:** PHBQ: at 1, 7 and 14 postoperative days |
| Stewart et al, 2019 | America | 4–12 years, ASA I-II | 102  (51 intervention and 51 control) | an age-appropriate game, 1 minute before parental separation to be continued through mask induction | midazolam 0.3 mg/kg (up to 20 mg max) p.o., 15 to 45 minutes before separation | **Anxiety:** mYPAS-SF: on admission (baseline), at time of parental separation and at mask induction  **Emergence delirium:** PAED: in the PACU on emergence and 15 minutes later for signs of emergence delirium  **Length of stay:** PACU arrival to emergence, PACU arrival to phase II, and PACU arrival to discharge home  **Behavior:** PHBQ-AS: at 7 and 14 postoperative days |
| Seiden et al, 2014 | America | 1-11 years, ASA I-II | 108  (57 intervention and 51 control) | age-appropriate video games, starting at the time of parental separation and concluding at induction | midazolam 0.5 mg/kg (up to 20 mg max) p.o., 15 to 45 minutes before entering the operating room | **Anxiety:** mYPAS: at parental separation and anesthetic induction  **Emergence delirium:** PAED: at emergence and 15 min after PACU admission  **Length of stay:** time-to-PACU arrival until awakening and time-to-PACU discharge  **Behavior:** PHBQ: at 7 and 14 postoperative days |
| Levay et al, 2023 | America | 3-5 years, ASA I-II | 99  (52 intervention and 47 control) | age-appropriate games, children had unlimited playtime, which varied from child to child, and continued through mask induction | midazolam 0.5mg/kg p.o., 15–20 min prior to arrival in the operating room | **Anxiety:** mYPAS: during mask induction  **Emergence delirium:** PAED: in PACU  **length of stay:** in PACU |
| ASA, American Society of Aneshesiologists; p.o., Peros, by mouth; p.r., Per rectum, by rectum; mYPAS, The Modified Yale Preoperative Anxiety Scale; mYPAS-SF, The Modified Yale Preoperative Anxiety Scale—Short Form; PHBQ, The Post Hospital Behavior Questionnaire; PHBQ-AS, The Posthospitalization Behavior Change Questionnaire for Ambulatory Surgery; PAED, The Pediatric Anesthesia Emergence Delirium; PACU, Post-Anesthesia Care Unit. | | | | | | |
